# Discovery and Annotation of Functional Chromatin Signatures in the Human Genome

**DOI:** 10.1371/journal.pcbi.1000566

**Published:** 2009-11-13

**Authors:** Gary Hon, Wei Wang, Bing Ren

**Affiliations:** 1 Bioinformatics Program, University of California at San Diego, La Jolla, California, United States of America; 2 Ludwig Institute for Cancer Research, University of California at San Diego, La Jolla, California, United States of America; 3 Department of Chemistry and Biochemistry, Center for Theoretical Biological Physics, University of California at San Diego, La Jolla, California, United States of America; 4 Department of Cellular and Molecular Medicine and Moores Cancer Center, UCSD School of Medicine, University of California at San Diego, La Jolla, California, United States of America; Weizmann Institute of Science, Israel

## Abstract

Transcriptional regulation in human cells is a complex process involving a multitude of regulatory elements encoded by the genome. Recent studies have shown that distinct chromatin signatures mark a variety of functional genomic elements and that subtle variations of these signatures mark elements with different functions. To identify novel chromatin signatures in the human genome, we apply a *de novo* pattern-finding algorithm to genome-wide maps of histone modifications. We recover previously known chromatin signatures associated with promoters and enhancers. We also observe several chromatin signatures with strong enrichment of H3K36me3 marking exons. Closer examination reveals that H3K36me3 is found on well-positioned nucleosomes at exon 5′ ends, and that this modification is a global mark of exon expression that also correlates with alternative splicing. Additionally, we observe strong enrichment of H2BK5me1 and H4K20me1 at highly expressed exons near the 5′ end, in contrast to the opposite distribution of H3K36me3-marked exons. Finally, we also recover frequently occurring chromatin signatures displaying enrichment of repressive histone modifications. These signatures mark distinct repeat sequences and are associated with distinct modes of gene repression. Together, these results highlight the rich information embedded in the human epigenome and underscore its value in studying gene regulation.

## Author Summary

Recent studies have observed that histone tails can be modified in a variety of ways. Analyzing a collection of 21 histone modifications, we attempted to determine what common signatures are associated with different classes of regulatory elements and whether they mark places of distinct function. Indeed, at promoters, we identified a number of distinct signatures, each associated with a different class of expressed and functional genes. We also observed several unexpected signatures marking exons that directly correlate with the expression of exons. Finally, we recovered many places marked by two distinct repressive modifications, and showed that they mark distinct populations of repetitive elements associated with distinct modes of gene repression. Together, these results highlight the rich information embedded in the human epigenome and underscore its value in studying gene regulation.

## Introduction

The genome sequence is a static entity defining the possible transcriptional output of every cell type in the human body [1]. By contrast, chromatin structure dynamically influences the transcriptional potential of each genomic loci in a particular cell. Over 100 different histone modifications are known to exist, and a single nucleosome can contain many modifications [2]. However, while the number of possible combinations of histone modifications far exceeds the number of nucleosomes in the human body, to date only a small number of histone modification patterns have been discovered [2].

Several classes of regulatory elements are marked by different chromatin signatures [3]–[5]. Notably, Heintzman et al recently observed distinct and predictive chromatin signatures at active promoters and enhancers [6],[7]. Numerous studies have also observed that slight variations in chromatin signatures can distinguish different states of the same regulatory element [3],[5]. For example, active promoters are generally marked by H3K4me3, repressed promoters by H3K27me3, and poised promoters by both marks [3]. Similarly, different chromatin signatures mark enhancers bound by different classes of transcription factors and co-activators [5]. In more recent studies, several chromatin signatures were also found at promoters [4], enhancers [4], and even exons [8]–[11] using genome-wide chromatin maps.

These observations prompted us to systematically examine the chromatin signatures that exist in known and putative regulatory elements in the human genome. Our goal is to explore whether other frequently occurring chromatin signatures exist, and whether specific functions are associated with these signatures. Focusing on 21 histone modifications mapped in CD4+ T cells [12], we find only a handful of distinct chromatin signatures at promoters, and that they correlate with gene expression. We then examine signatures spanning almost 50,000 regions in the human genome that are distal to previously annotated regulatory sites. We recover 7 distinct chromatin signatures, some containing enrichment of H3K36me3 that has been recently linked to marking exons [8]. Upon further inspection, we observe that H3K36me3 is most strongly enriched at a well-positioned nucleosomes located at the 5′ ends of exons. We also find that stronger enrichment of H3K36me3 correlates with increased exon usage in alternatively spliced genes. Finally, we recover two distinct chromatin signatures rich in repressive histone modifications marking distinct regions of the genome, that are associated with different modes of gene repression.

## Results

### Chromatin signatures distinguish different classes of expressed promoters

We hypothesize that loci sharing common regulatory functions may share similar chromatin signatures. To systematically identify chromatin signatures genome-wide, we examine different classes of regulatory loci in turn. These loci may contain chromatin signatures, but they may not be aligned or even oriented in the same direction. We therefore apply an unbiased clustering and alignment method called ChromaSig [5] (see Methods) to find over-represented chromatin modification patterns spanning these loci while simultaneously aligning and orienting their enrichment profiles, focusing on histone modification maps profiled recently in CD4+ T cells [12]. As a proof of principle that this approach yields biologically significant results, we first studied promoters.

**Figure 1:**
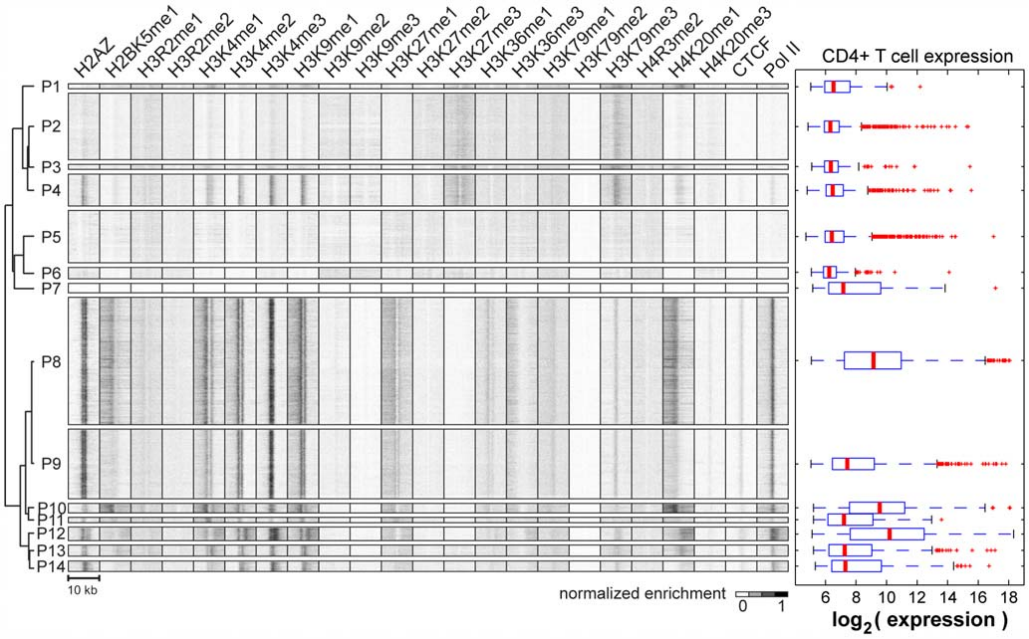
Distinct chromatin signatures spanning Refseq promoters. (left) Applying ChromaSig to the histone modifications near 20,389 Refseq promoters recovers 14 frequently-occurring chromatin signatures spanning 18,533 promoters. The heat map represents the enrichment of H2AZ, 20 histone modifications, CTCF, and RNA polymerase II in the 10-kb region surrounding each promoter. To organize these clusters visually, we performed hierarchical clustering on the average profiles using a Pearson correlation distance metric. (right) Gene expression data for CD4+ T cells measured from a previous study [14], and re-visualized here for the different classes of promoters. Shown are the distributions of gene expression level over promoters with different chromatin signatures. Red horizontal lines indicate the median, the box extends to the lower and upper quartiles, the whiskers extend to 1.5 times the inter-quartile range, and red “+” symbols are outliers.

While chromatin signatures at promoters have been studied extensively, we still do not have a complete picture of all the distinct, commonly occurring chromatin signatures spanning all promoters. As such, our understanding of how different signatures relate to gene expression is incomplete. To address this, we apply ChromaSig to the chromatin modifications near the set of manually annotated promoters defined in the Refseq database [13]. We recover 14 clusters spanning 18,533 promoters (**Fig. 1**, **Table 1** **, Table S1**). Promoters in the same cluster share a common chromatin signature, and the chromatin signatures of different clusters are distinct in apparent or subtle ways. For example, the P4 cluster contains strong enrichment for various H3K4 methylations while P2 lacks these modifications. On the other hand, P9 and P12 clusters contain enrichment for the same chromatin modifications, but the pattern of enrichment is different, with P12 containing enrichment over a noticeably wider region. It is also evident that there is a high level of redundancy of histone modifications at promoters. Notably, H2AZ, H3K4me1, H3K4me2, H3K4me3, and H3K9me1 are either all found together or all absent together at promoters, consistent with recent findings [4].

**Table 1:** Summary of promoter chromatin signatures P1–14.

Cluster	Size	Chromatin features	P (CpG)*	Top GO Biological Process**	
P1	208	H3K27me3, H4K20me1	<1E-16	multicellular organismal dev	anatomical structure dev
P2	2896	H3K27me3	1	multicellular organismal dev	neurological system proc
P3	204	H3K27me3	<1E-16	multicellular organismal dev	anatomical structure dev
P4	1379	H3K4me3, H3K27me3	<1E-16	multicellular organismal dev	anatomical structure dev
P5	2270	none	1	sensory perception	neurological system proc
P6	487	none	1	sensory perception	neurological system proc
P7	392	none	1	None	
P8	5535	H3K4me3, H4K20me1, H2BK5me1, H3K36me3	<1E-16	primary metabolic proc	cellular metabolic proc
P9	3035	H3K4me3	<1E-16	primary metabolic proc	cell cycle
P10	409	H3K4me3, H4K20me1, H2BK5me1, H3K36me3	<1E-16	regulation of biological proc	regulation of cellular proc
P11	219	H3K4me1	1	None	
P12	575	H3K4me3, H4K20me1, H2BK5me1, H3K36me3	<1E-16	primary metabolic proc	biopolymer metabolic proc
P13	472	H3K4me3, H4K20me1	<1E-16	multicellular organismal dev	cell differentiation
P14	452	H3K4me3	8.43E-04	None	

* P(CpG) is the hypergeometric probability of finding more CpG-marked promoters than observed, as compared to the background distribution of all promoters. ** Selected Gene Ontology terms from the Biological Processes ontology significantly enriched with Benjamini-corrected p-value of 0.001. Abbreviations: dev, development; proc, process.

Previous studies have shown that there are at least three different classes of chromatin signatures at promoters: actively transcribed promoters marked by H3K4me3 but not H3K27me3, inactive promoters marked with H3K27me3 but not H3K4me3, and bivalent promoters having both these marks [3]. ChromaSig recovers all three of these previously known chromatin signatures: P8–14 have the active chromatin signature, P2 contains the repressed chromatin signature, and P4 has the bivalent signature. In agreement with a previous study, we observe that 1379 (7.4%) promoters in human CD4+ T cells are bivalent, compared to similar numbers in the differentiated mouse embryonic fibroblasts (8.6%) but lower than that found in undifferentiated mouse embryonic stem cells (15.2%).

Next, we wondered if different signatures correspond to different gene expression activities. On the basis of gene expression [14], we observe essentially three super-classes of promoters: P1–7 are generally inactive in CD4+ T cells, P9,11,13,14 show intermediate expression, and P8,10,12 are most highly expressed (**Fig. 1**). Promoters with repressed and bivalent chromatin signatures are generally expressed at low levels, while promoters with active chromatin signatures have intermediate to high levels of gene expression. Consistent with the high expression levels, P8, P10, and P12 also display the most enrichment of the elongation chromatin mark H3K36me3 (**Fig. 1**) [12],[15]. Interestingly, we observe chromatin signatures of varying widths of H3K4me3 immediately surrounding transcription start sites. We find that clusters with larger H3K4me3 widths tend to correspond to higher gene expression. For example, by visual inspection the average width in P12 is larger than P10, which is in turn larger than P8, and which is larger than P9. Strikingly, median gene expression levels also decrease in the same order.

CpG islands often mark the promoters of house-keeping genes that are ubiquitously expressed [16],[17]. Strikingly, we observe that each distinct chromatin signature contains promoters that are either significantly enriched or depleted of CpG islands (**Table 1**). Nine of the 14 recovered clusters, containing 66% of all promoters, are significantly enriched in CpG islands (hypergeometric p-value of 1E-3). The majority of these CpG-enriched promoters (75%) belong to clusters P8, P9, and P12 containing the strongest enrichment of H3K4me3. As expected from the high CpG content, these promoters are also significantly enriched in Gene Ontology (GO) [18],[19] terms relating to ubiquitous processes such as metabolism and the cell cycle. Another 11% of the CpG-rich promoters are in cluster P4 containing bivalent promoters marked by H3K4me3 and H3K27me3. Consistent with previous studies [3],[20], these promoters are enriched in GO terms relating to human development.

In contrast, clusters P2,5,6,7,11 spanning 34% of all promoters are significantly depleted of CpG islands. Nearly half of these promoters are marked by H3K27me3 but not H3K4me3 in cluster P2. Consistent with previous studies suggesting these promoters are inactive [3],[20], many of these associated genes are enriched in GO terms relating to development and neurological processes, which are unrelated to T-cell function. Interestingly, P2 and P4 both mark repressed genes involved in development, but with distinct sequence context and chromatin signatures. P5 and P6 are the most CpG depleted clusters, and are not enriched in any histone modifications studied here. The corresponding genes are lowly expressed, and are enriched in GO terms unrelated to T-cells such as secretion and sensory perception [19]. Finally, P11 is the only CpG-poor cluster enriched with activating chromatin marks. Consistent with the notion that the corresponding genes are likely involved in cell-type specific processes [20], these genes are generally more highly expressed than other CpG poor promoters, and include T-cell specific genes such as cathepsin W, which regulates T-cell cytolytic activity, the T-cell specific protease granzyme A, as well as several lymphocyte antigens including LY86, CD68, and CD79A.

Together, these results show that ChromaSig can reliably detect distinct chromatin signatures at promoters with unique functional specificities.

### Distinct chromatin signatures at known regulatory elements

While transcriptional regulation occurs at the level of promoters, it is also clear that the action of promoter-distal regulatory elements is essential to controlling gene expression [1]. Like promoters, the activity of these regulatory elements is likely dependent on chromatin structure. To determine what chromatin signatures exist at distal regulatory elements, we apply ChromaSig to several classes of regulatory elements in turn: enhancers, insulators, Refseq 3′ ends, and DNase I hypersensitive sites.

#### Enhancers

When active, enhancers are bound by transcription factors and co-activators to increase gene expression at promoters [21],[22]. Previously, we observed that enhancers are marked by strong enrichment of H3K4me1 and weak if any enrichment of H3K4me3, allowing us to develop a computational strategy to identify enhancers using this chromatin signature [6]. Applying this method to the genome-wide profiles of H3K4me1 and H3K4me3 in CD4+ T cells [12], we predict 32,237 promoter-distal enhancers (see Methods). To validate these enhancer predictions, we compare to two hallmarks of enhancers: DNase I hypersensitivity and sequence conservation. Almost half (44.5%) of the enhancer predictions are within 1-kb of a DNase I hypersensitive site [23], and about three-fourths of the predictions are recovered by some combination of hypersensitivity and conserved DNA sequence elements from the PhastCons database [24].

We have previously observed in 1% of the human genome (the ENCODE regions) that different variations of chromatin modifications exist at enhancers [25]. To assess if this is true on a global scale, we apply ChromaSig to align and cluster these predicted enhancers over the entire panel of chromatin modifications. This reveals 11 distinct chromatin signatures, all of which contain stronger enrichment for H3K4me1 than H3K4me3 (**Fig. S4**, **Table S2**). Like promoters, there also appears to be much redundancy of chromatin modifications at enhancers. For example, all chromatin signatures generally share enrichment for H2BK5me1, H3K4me2, H3K9me1, H3K27me1, and H3K36me1. Interestingly, the chromatin marks H2A.Z and H4K20me1 appear to be inversely correlated: E1-5 are enriched in H2A.Z but not H4K20me1, E6 has enrichment of both marks, and E7–11 are enriched in H4K20me1 but not H2A.Z.

#### Insulators

CTCF is an insulator binding protein in mammals, and when bound prevents enhancers from interacting with promoters, thereby preventing activation [26]. Barski et al mapped CTCF binding in CD4+ T cells [12], and application of the Model-based Analysis of ChIP-Seq (MACS) peak finder reveals 27,110 CTCF binding sites genome-wide (see Methods) [27]. To focus on novel chromatin signatures, we apply ChromaSig to the 17,328 CTCF sites distal to (at least 2.5-kb) Refseq TSSs and predicted enhancers, revealing seven distinct signatures (**Fig. S5**, **Table S3**). The only consistent feature of CTCF binding sites is enrichment of H2A.Z, consistent with previous observations [28]. However, unlike the patterns observed at promoters and enhancers, enrichment for other chromatin marks at CTCF binding sites is generally weak, suggesting that the remaining panel of chromatin marks do not functionally compliment CTCF. The exceptions are C4 and C5, which contain enrichment of H3K4me3 and RNA Pol II, suggesting that these CTCF binding sites may fall within promoters not yet annotated in the Refseq database.

#### Refseq 3′ ends

Transcription of pre-mRNA stops at the 3′ end of the gene. To find chromatin signatures at this genomic feature, we apply ChromaSig to 16,703 Refseq gene 3′ ends distal to Refseq 5′ ends [13]. We recover 12 distinct chromatin signatures. Like CTCF binding sites, enrichment of chromatin marks at Refseq 3′ ends is generally weak. In agreement with Barski et al [12], the most consistent feature found at the majority of 3′ ends is enrichment of H3K36me3, found in T1–7 (**Fig. S6**, **Table S4**). However, chromatin signatures at 3′ ends are not as well aligned as those at promoters, suggesting that these chromatin signatures may occur at some other genomic feature near 3′ ends, or that the 3′ ends are not as well annotated as promoters.

#### DNase I hypersensitive sites

Recently, Boyle et al mapped nearly 100,000 DNase I hypersensitive sites genome-wide in CD4+ T cells using DNase-Seq [23]. Since DNase I hypersensitivity is a hallmark for active regulatory loci, we expect to find chromatin signatures at these sites. Applying ChromaSig to the 31,824 DNase I hypersensitive sites distal to Refseq TSSs, predicted enhancers, and CTCF binding sites, we recover 13 clusters (**Fig. S7**, **Table S5**). Clusters D1–D2 are only enriched in H3K27me1 and H3K36me3, resembling gene 3′ ends. Several signatures D3–10 display characteristic enrichment of H3K4me1/2/3, which we have observed at promoters and enhancers. These may be novel promoters or enhancers missed by the enhancer prediction method. For example, D3,6,9,10 are clusters with the strongest enrichment of H3K4me3, and 31.2% of these loci are recovered by multiply-occurring CAGE tags [29], an almost 4-fold enrichment as compared to an expected recovery of 7.9% over random loci. The majority of DNase I sites D11–13 contain no noticeably strong enrichment of any chromatin mark, suggesting either that there are no other major classes of epigenetically-marked regulatory elements in the human genome or that they are marked by modifications not studied here.

### Several clusters of enhancers correlate with gene activity

In eukaryotes, control of gene expression is a complex process involving the coordinated action of a wide assortment of genomic regulatory elements. Of the five classes of genomic regulatory elements examined here, the ones least studied and perhaps most important to controlling gene expression are enhancers and DNase I hypersensitive sites. To examine the potential regulatory roles of these genomic loci, we measure the enrichment of these loci near different classes of expressed genes as defined by the 14 clusters of promoter chromatin signatures (**Fig. 1**).

When a CTCF-bound insulator falls between a promoter and enhancer, the enhancer is blocked from activating the promoter [26]. As this mechanism may also apply to regulatory elements outside of enhancers, we partition the genome into CTCF-defined blocks and determine enrichment of chromatin signatures having promoters in the same CTCF-defined block (**Fig. S8**). At a large scale, we observe that inactive promoters P1–6 generally lack enrichment for all the chromatin signatures cataloged here. In contrast, CTCF-defined domains containing active promoters P8–14 are enriched in numerous chromatin signatures. Strikingly, different classes of promoters are enriched in different classes of enhancers. For example, the two most highly expressed clusters P10 and P12 are uniquely enriched in E6–11. These enhancers are distinguished from other enhancer classes by strong enrichment of H3K9me1 and H4K20me1, indicating that these chromatin marks may be an indicator of enhancer activity. Of these enhancers, the class that most distinguishes highly active promoters from all other promoters is E9. This cluster may contain the most active enhancers, and its chromatin signature may be a general mark for highly active enhancers. In general, we observe weaker enrichment of the DNase I hypersensitive clusters within CTCF-defined blocks containing highly expressed promoters, with the exception of D6–8 which are likely enriched in novel promoters and enhancers missed by the enhancer prediction method.

### Distinct chromatin signatures distal to known regulatory regions

**Figure 2:**
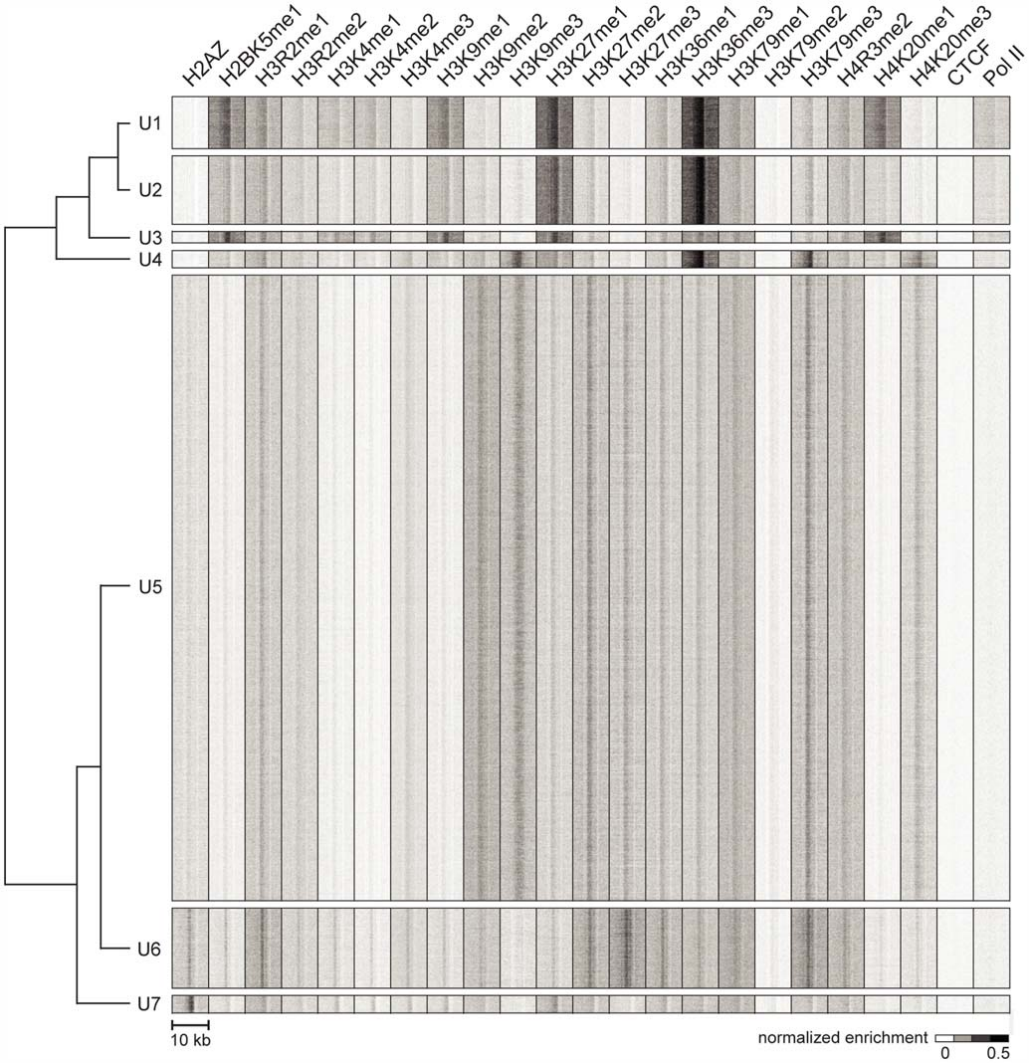
Distinct chromatin signatures spanning genomic loci distal to known regulatory elements. We identified 50,183 genomic loci with strong ChIP enrichment of histone modifications but distal to promoters, gene 3′ ends, DNase I hypersensitive sites, CTCF binding sites, and predicted enhancers. Applying ChromaSig to these loci reveals seven clusters U1–7 spanning 47,874 loci. The heat map represents the enrichment of H2AZ, 20 histone modifications, CTCF, and RNA polymerase II in the 10-kb region surrounding each locus. To organize these clusters visually, we performed hierarchical clustering on the average profiles of each ChromaSig cluster, using a Pearson correlation distance metric (left).

Having observed chromatin signatures at regulatory elements including promoters and enhancers, we next ask if other chromatin signatures exist that mark loci distal to known regulatory elements. By definition, places in the genome with chromatin signatures contain enrichment of histone modifications. Therefore, we identify 85,318 loci with strong ChIP enrichment of histone modifications, of which 50,183 are distal to promoters [13], gene 3′ ends [13], DNase I hypersensitive sites [23], CTCF binding sites [12], and sites containing an enhancer chromatin signature [6],[7]. Applying ChromaSig to these sites, we recover 7 frequently-occurring chromatin signatures, named U1–7 (for unannotated clusters 1 to 7), spanning 47,874 loci (**Fig. 2** **,** **Table 2**, **Table S6**). The recovered signatures are distinct from the previously defined H3K4me3-rich promoter and H3K4me1-rich enhancer signatures [3],[6]. Compared to chromatin signatures from randomly aligned and oriented loci, the chromatin signatures observed are significantly better aligned than expected by chance (p-values ranging from 10^−18^ to <10^−300^) (**Table S7**).

**Table 2:** Summary of chromatin signatures U1–7.

Cluster	Number	Chromatin features	Association
U1	2845	H3K36me3, H2BK5me1, H4K20me1	Exons
U2	3742	H3K36me3	Exons
U3	615	H2BK5me1, H4K20me1	?
U4	961	H3K36me3, H3K9me3	Exons
U5	34368	H3K9me3, H3K27me3	Repressed regions
U6	4394	H3K27me3	Repressed regions
U7	949	H2AZ	?

The most prominent chromatin feature of these clusters is H3K36me3, known to mark the 3′ ends of genes [12] and more recently exons [8], and it is enriched at U1, U2, and U4 clusters. The largest clusters recovered, U5 and U6, both contain enrichment of known repressive chromatin modifications including H3K9me2, H3K9me3, H3K27me2, and H3K27me3 [12].

### Chromatin signatures mark exon 5′ ends

To gain an understanding of potential functions associated with the above frequently-occurring novel chromatin signatures, we compare the loci bearing each signature to genomic annotations.

H3K36me3 is known to be enriched within the body of transcriptionally active genes [30],[31], notably towards the 3′ ends [12]. But since all the clustered loci are distal to gene 3′ ends, the H3K36me3-rich clusters must be marking another genomic feature. Noticing that the vast majority of loci in U1–4 are intragenic (**Fig. S9**), we ask if these sites are biased towards exons or introns. We observe that 57.9% of U1 sites and 63.8% of U2 sites are either inside exons or within 1-kb of exon ends, while at random only 26% of the genic regions of the genome match these criteria. To see if H3K36me3 marks exons, we examine the enrichment of this chromatin mark at exons (**Fig. S1**). To examine only those exons unambiguously marked by a chromatin signature, we only consider an exon if it is the only exon within 1-kb of a cluster locus. We observe a striking enrichment of H3K36me3 at the 5′ ends of exons unambiguously marked by U1, U2, and U4. This enrichment decreases sharply upstream of the 5′ end, but more gradually into the exon body. This observation also holds for exons larger than 1-kb (**Fig. S2**), indicating that the result is not biased by the relatively small exon sizes in the human genome [32]. These results suggest that the clusters with strong H3K36me3 enrichment mark exon 5′ ends.

### H3K36me3 reflects exon expression levels

**Figure 3:**
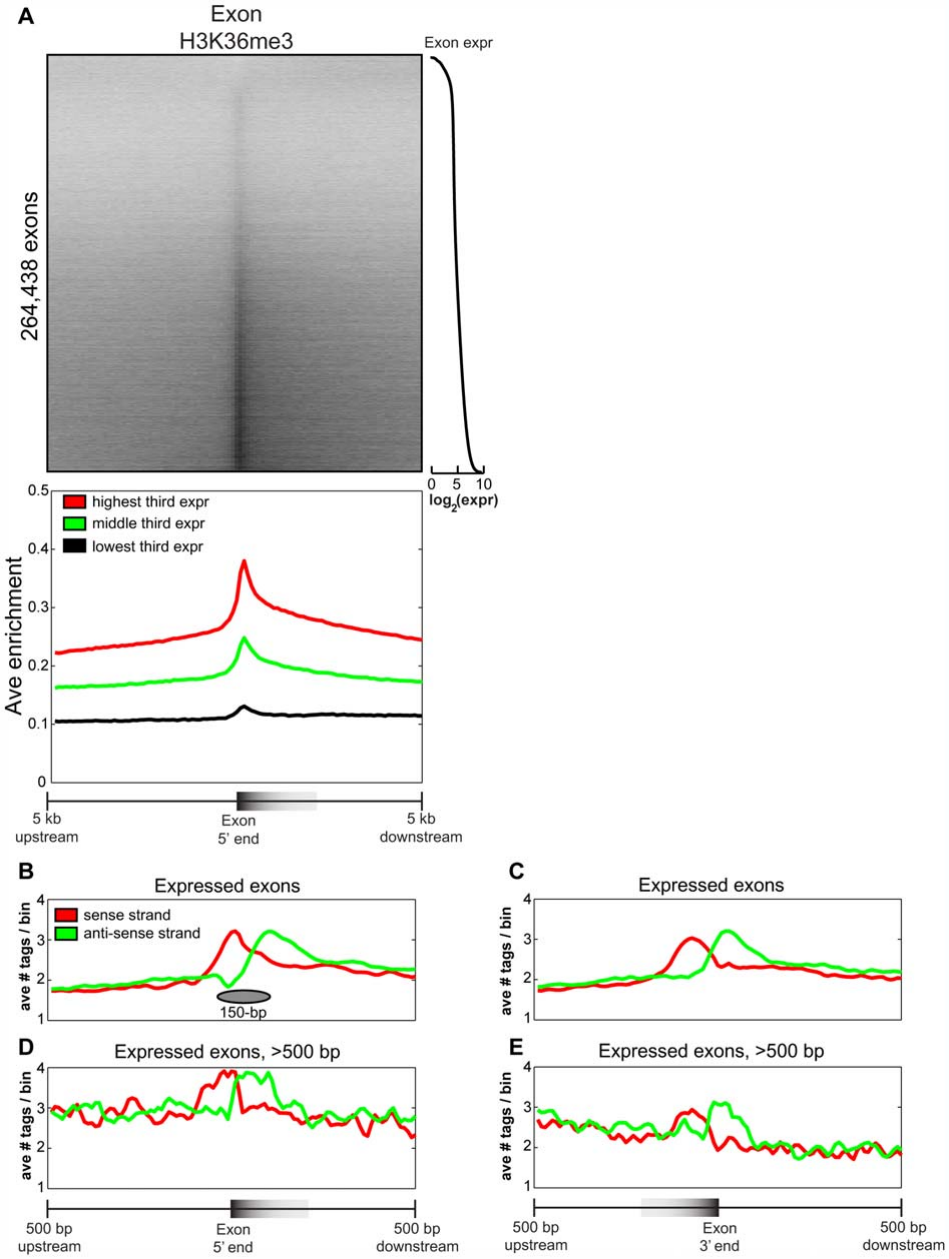
H3K36me3 marks exon 5′ ends and is a global mark of expression. (A) The top panel is a heat map of H3K36me3 enrichment at all human exons, sorted by exonic expression (right). The bottom panel is the average H3K36me3 enrichment profile of the lowest, middle, and highest third of expressed exons from the top panel. The distribution of H3K36me3 reads within ±500 bp of exon (B) 5′ ends and (C) 3′ ends of the top 50% expressed exons in the human genome. In red are reads on the sense strand in the direction of transcription, and in green are anti-sense reads. A schematic of a positioned a nucleosome is shown. (D–E) As in (B–C), but focusing on expressed exons longer than 500 bp.

Having observed H3K36me3 at a handful of exons, we next ask if this chromatin mark is a global indicator of exon expression. First, we examine the enrichment of clusters U1–4 within the gene bodies belonging to the promoters in clusters P1–14. Indeed, we find that clusters U1–4 are enriched within the gene bodies of highly expressed genes belonging to promoter classes P8–P14, but are depleted in the gene bodies of inactive promoters in other classes (**Fig. S8**). Next, profiling H3K36me3 at a catalog of more than 250,000 distinct exons [33], we observe that the majority of exons (72.6%) have more than two-fold enrichment for H3K36me3 tags than neighboring introns (**Fig. 3A**). In the direction of transcription, H3K36me3 enrichment increases sharply at the 5′ end of the exon, and decreases more gradually in the body of the exon, in agreement with our previous observations. In contrast, neighboring introns show no such chromatin signature (**Fig. 3** **, S10**). The presence of this chromatin mark also correlates strongly with exonic expression (**Fig. 3**), as measured previously by exon expression arrays in CD4+ T cells [34]: highly expressed exons having more H3K36me3 enrichment than lowly or moderately expressed exons. Altogether, these results suggest that H3K36me3 is a general mark of exon expression.

### Stable nucleosome structure at exon 5′ ends

Recently, it has also been observed that H3K36me3 marks exons in various eukaryotes, though the modification was found to be biased toward the 3′ ends of exons [8]. To resolve this discrepancy, we take advantage of a unique feature of ChIP-Seq technology, which sequences short directional reads directly upstream and downstream of the genomic DNA bound by the protein of interest, allowing clear distinction between sense and anti-sense reads. This information can be used to offer unprecedented resolution of *in vivo* binding locations of the immunoprecipitated protein [27],[35]. We can also use this information to more finely resolve nucleosome structure at exons. Examining the distribution of H3K36me3 tags near the top 50% expressed human exons, we observe that reads on the sense strand peak at the 5′ ends of exons, whereas reads on the anti-sense strand peak about 150 base pairs downstream (**Fig. 3B**). These results suggest that a well-positioned nucleosome modified by H3K36me3 exists at the 5′ ends of expressed exons, and consistent with this conclusion the spacing between sense and anti-sense peaks is roughly the size of a nucleosome.

In addition to exon 5′ ends, it also appears that the 3′ ends of expressed exons have well-positioned nucleosomes (**Fig. 3C**). But given that a typical nucleosome wraps between 145 and 147 bp of DNA [36], which is roughly the same size as the average human exon at 145 bp [32], it is difficult to conclude from these observations whether the nucleosomes harboring H3K36me3 are more fixed towards exon 5′ or 3′ ends. To resolve this issue, we re-examine the distribution of H3K36me3 reads, but focus on expressed exons larger than 500 bp (**Fig. 3D–E**). Again, we observe sense and anti-sense peaks at exon 5′ ends indicative of well-positioned modified nucleosomes, followed by a decrease of H3K36me3 enrichment on both strands in the direction of transcription. However, we also find similar but weaker peaks on both strands at exon 3′ ends, with the sense strand peaking about a nucleosomal distance upstream of the anti-sense strand (**Fig. 3E**). Thus, we conclude that the nucleosomes harboring H3K36me3 are found at both 5′ and 3′ ends of exons, but the enrichment is stronger at the 5′ ends. To test this conclusion more globally over a larger collection of exons, we also examine the enrichment of H3K36me3 along the exon body as a function of exon length. Indeed, as exon length increases, we observe enrichment of H3K36me3 at 5′ and weaker enrichment at 3′ exon ends, separated by the exon body lacking enrichment (**Fig. S11**).

### H3K36me3 correlates with alternative splicing

**Figure 4:**
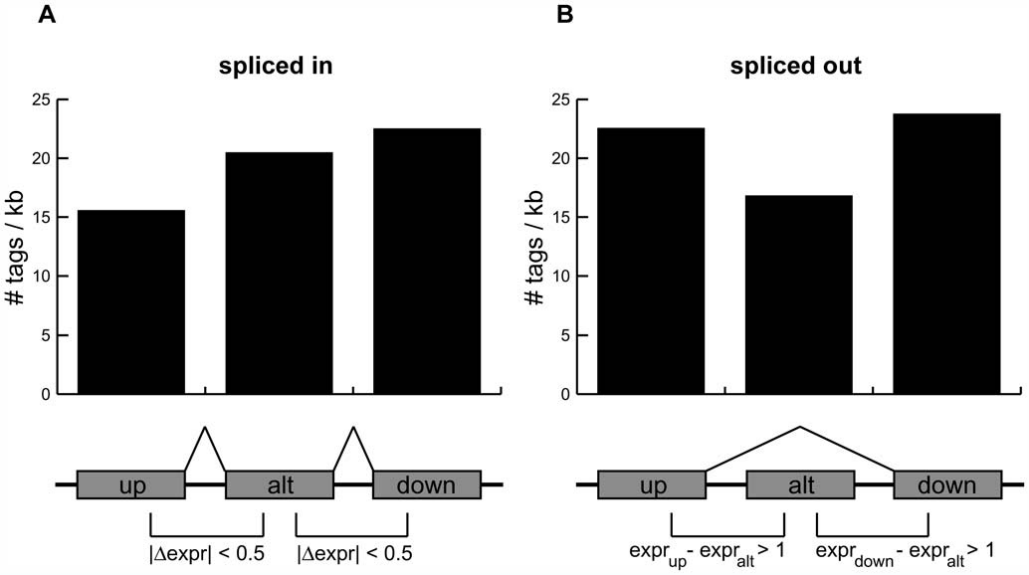
H3K36me3 enrichment correlates with alternative splicing. The number of H3K36me3 reads per kilobase for exons near alternatively spliced cassette exons that are (A) spliced in or (B) spliced out. A cassette exon is defined to be spliced in if the difference in expression between it and its immediate upstream and downstream exons is less than 0.5 on a log2 scale. A cassette exon is defined to be spliced out if both upstream and downstream exons are at least 2-fold more expressed (1.0 on a log2 scale).

As H3K36me3 at the 5′ ends of exons is a global mark of exon expression, we next wondered if the presence of this mark correlates with alternative splicing. A previous study found that the density of H3K36me3 at canonical exons is higher than that at alternative exons in mice [8]. As this observation did not incorporate expression information but instead relied on static exon definitions, the question of whether the presence of H3K36me3 correlates with exonic splicing in humans remains unanswered. To answer this question, we investigate alternative splicing on a global scale by focusing on a list of 13,434 exons known to be alternatively spliced as cassette exons (UCSC Genome Browser “knownAlt” track) [37]. We examine two sets of transcripts using exonic expression information. The “spliced in” set consists of cassette exons expressed at levels similar to neighboring upstream and downstream exons (|Δexpr| = 0.5), and thus are likely to be included in a mature transcript. In contrast, the “spliced out” set consists of cassette exons expressed at lower levels than both upstream and downstream exons, and are likely excluded from the mature transcript (expr_up,down_−expr_alt_>1). For spliced in exons, we observe that the enrichment of H3K36me3 increases gradually from upstream to alternatively spliced to downstream exons (**Fig. 4A**), consistent with previous results showing a 3′ bias in this chromatin mark [12]. However, H3K36me3 is noticeably depleted at spliced out exons as compared to both upstream and downstream exons (**Fig. 4B**). These results suggest that, on a global scale, the presence of H3K36me3 at alternatively spliced exons correlates with inclusion of the exon in transcripts.

In agreement with these observations, we find that exons marked by U1 or U2 are preferentially included in mature mRNAs (p_U1_ = 1.65E–26, p_U2_ = 5.94E–43, Wilcoxon rank sum test) (**Fig. S3**). U3, which contains no H3K36me3 enrichment (**Fig. 2** **, S1**), is a negative control containing no preference of exon inclusion. Interestingly, exons marked by U4, which are enriched in the repressive H3K9me3 modification, are preferentially excluded from mature mRNAs (p_U4_ = 6.67E–4, Wilcoxon rank sum test). Taken together, these results suggest that several distinct chromatin signatures are found at exon 5′ ends, that some signatures mark exons for preferential inclusion, and others for preferential exclusion. These different functional specificities may be attributed to specific differences in chromatin signatures (see Discussion).

### H2BK5me1 and H4K20me1 mark highly expressed 5′ exons

**Figure 5:**
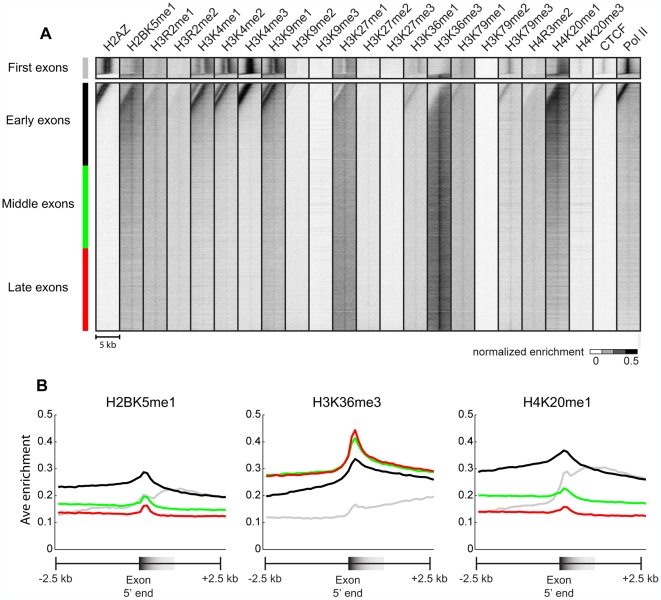
H2BK5me1 and H4K20me1 mark early exons. (A) Shown is a heat-map representing the enrichment of various modifications and factors in a 5-kb region surrounding the top third expressed exons. The exons are separated into (top) first exons and (bottom) non-first exons, and are then sorted by distance from the transcription start site. Non-first exons are further subcategorized into early, middle, and late exons. (B) The average profiles for (left) H2BK5me1, (middle) H3K36me3, and (right) H4K20me1 for first, early, middle, and late exons.

Our initial scan revealed several classes of chromatin signatures marking exons, the largest of which are U1 and U2. Both of these contain enrichment for H3K36me3, but U1 contains stronger enrichment for H2BK5me1 and H4K20me1. This latter modification is known to be localized both at promoters and intragenic regions downstream of the promoters, with enrichment fading in the gene body [12]. These observations raise the possibility that exons marked by U1 are exons closer to promoters (5′ exons) while U2 are exons closer to the 3′ ends of genes (3′ exons). To test this hypothesis, we partition the highly expressed exons above into first and non-first exons. Non-first exons are further subcategorized into early, middle, and late exons based on distance from the transcription start site (TSS). We then examine the enrichment of histone modifications near these different classes of exons (**Fig. 5**). As expected, first and early exons, which are closest to TSSs, are all highly enriched in promoter modifications including H3K4me1, H3K4me2, and H3K4me3. In addition to H3K36me3, it is clear that there is also a general peak of H2BK5me1 and H4K20me1 enrichment at exons. This enrichment is most pronounced in 5′ exons compared to first, middle, and 3′ exons. In addition, we also observe that 5′ exons, while still marked by H3K36me3, have weaker enrichment of this mark compared to mid or 3′ exons, but is clearly more enriched than the first exon. H3K36me3 enrichment increases with increasing distance from the TSS, consistent with above results (**Fig. 4A**) and previous observations [12]. These results provide additional evidence for various chromatin modifications marking distinct exons in the human genome.

### Distinct classes of repressive chromatin signatures

In addition to chromatin signatures U1–4, ChromaSig also identifies two new chromatin signatures, U5–6, having strong enrichment of repressive histone modifications (**Fig. 2**). Consistently, these signatures are not found near highly expressed genes but are enriched near repressed genes (**Fig. S8**). These two chromatin signatures are distinct, with U5 having stronger enrichment of repressive modifications H3K9me2 and H3K9me3. This subtle difference prompted us to ask if these signatures mark distinct regions of the genome. Indeed, we find that only 23.3% of U5 loci are intragenic, a notable depletion over the expected value of about 40% (**Fig. S9**). In contrast, U6 loci are closer to the expected value at 36.3% intragenic.

**Figure 6:**
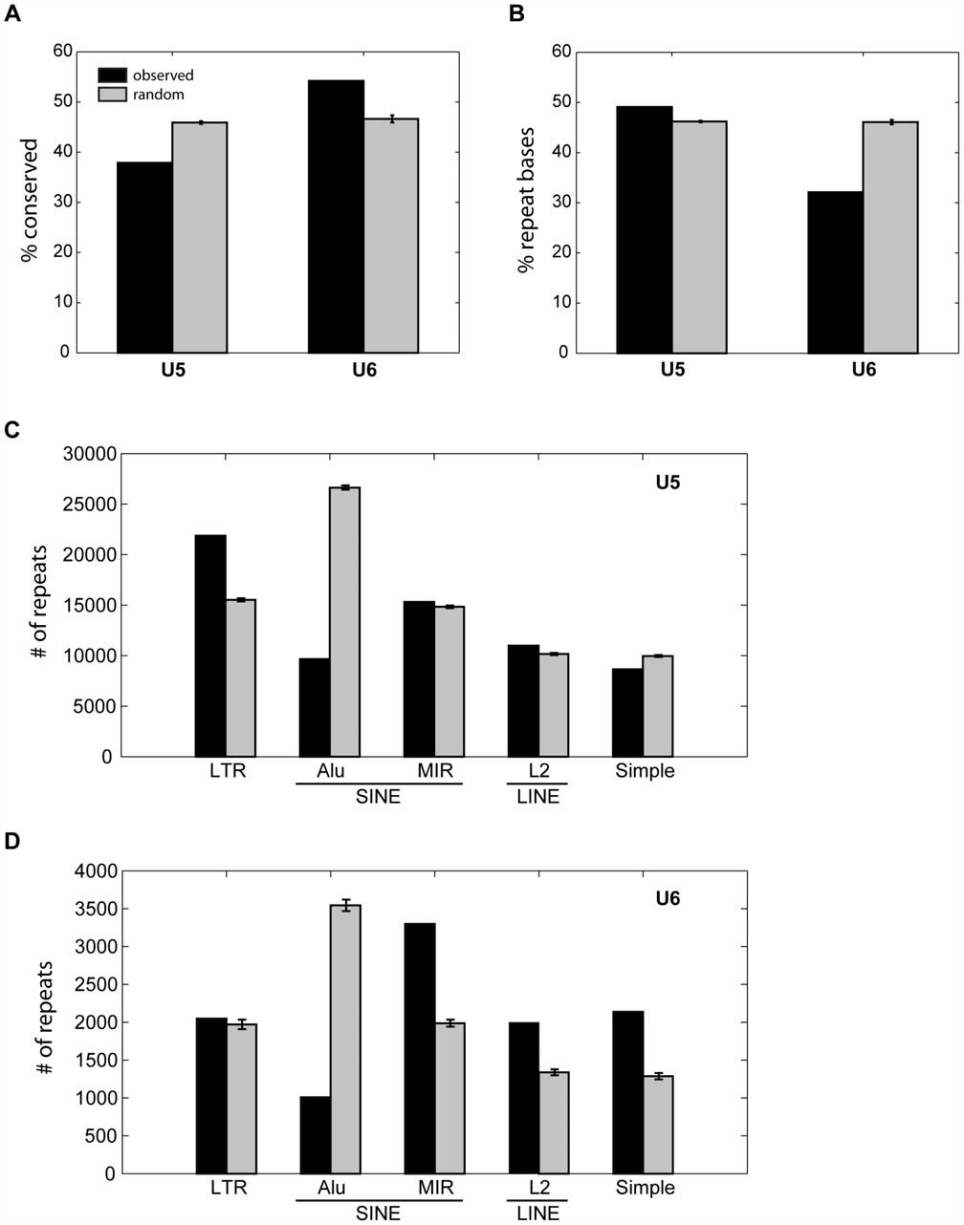
U5 and U6 mark distinct sequences of the genome. (A) The percentage of loci in U5 and U6 within 1-kb to an evolutionarily conserved PhastCons element. (B) The average percentage of bases ±1 kb around each locus that are masked by RepeatMasker. (C–D) The number of repeat elements within ±1 kb of each locus in (C) U5 and (D) U6. Black indicates the observed value while grey indicates the expected value over random sites. The error bars indicate ±1 standard deviation. LTR, long terminal repeat; simple, simple repeat.

Additional analysis suggests that the sequences underlying U5 and U6 fragments are associated with distinct properties. First, we compare to the PhastCons database containing over 2 million conserved elements in the human genome conserved over 28 mammalian genomes [24]. We find that U5 loci are significantly depleted of conserved elements (p = 7.12E–182) while U6 is significantly enriched (p = 2.09E–26) (**Fig. 6A**). Given that repressive histone modifications have been known to mark repetitive regions of the genome [38] which are highly lineage-specific [32], the low conservation of U5 loci may be explained by enrichment for repetitive sequences. To test this hypothesis, we use RepeatMasker [39] to define repetitive bases within ±1-kb from each locus in U5–6. Indeed, 49.1% of U5 bases are repetitive, as compared to 32.1% of U6 bases (**Fig. 6B**), suggesting that these two clusters may harbor different classes of sequences. To pursue this further, we next ask if the classes of repeats found in U5 are different from those found in U6. Counting the repetitive elements found within ±1-kb of each locus (**Fig. 6C,D**), we find that U5 is significantly enriched for long terminal repeats (LTR) (p<1E–300, Z-score = 39.7), while U6 is neither enriched nor depleted. For the SINE family of repeats, while both clusters are significantly depleted in Alu repeats (p_U5_<1E–300, Z_U5_ = 81.5; p_U6_ = 4.76E–245, Z_U6_ = 33.4), only U6 is notably enriched in MIR repeats (p = 2.31E–177). Similarly, L2 LINE repeats and simple repeats are notably more enriched in U6 loci than U5 loci. These results suggest that U5 and U6 have different genic distributions and mark distinct sequences of the genome.

### U5 and U6 mark different domains of gene repression

**Figure 7:**
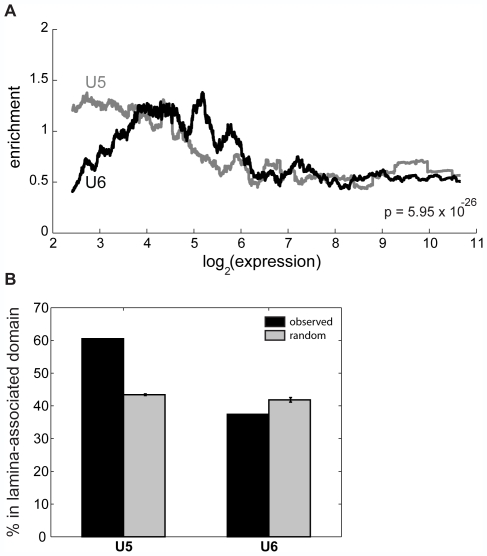
U5 and U6 mark distinct expression domains of the genome. (A) Enrichment of U5 and U6 loci as a function of expression for genes in the same domain. We counted the number of U5 and U6 loci within the CTCF-defined domains containing human promoters, assessed enrichment as compared to that expected over random sites, and averaged over a 1000-promoter sliding window to create each profile. The signed rank p-value is indicated. (B) The percentage each cluster within lamina-associated domains, previously mapped in Tig3 human lung fibroblasts (black), as compared to random sites (grey). The error bars indicate ±1 standard deviation.

We next examine whether the different genic distributions and sequence preferences of U5 and U6 relate to gene expression. It is thought that the genome is organized into different domains of transcriptional activity, with the insulator binding protein CTCF defining the boundaries of these domains [26]. Therefore, we partition the genome into CTCF-defined domains and determine the enrichment of U5 or U6 loci in these domains as a function promoter activity. The distributions of U5 and U6 enrichment are significantly different (p = 5.95E–26, paired Wilcoxon signed rank test) (**Fig. 7A**): U5 is more enriched than U6 in domains containing the most repressed genes (log expression <4), while domains containing genes more expressed (log expression between 5 and 6) have higher enrichment of U6 loci than U5 loci. For moderately and highly expressed genes (log expression >6), the enrichment of both U5 and U6 loci are depleted relative to random. We next investigate the localization of U5 and U6 with respect to the distinct promoter classes P1–14. We find that U5–6 are in general depleted near moderately and highly expressed promoters P8–14. In contrast, U5 and U6 are enriched near distinct classes of repressed genes. U6 is enriched in CTCF blocks containing P1 and P3 compared to U5 (**Fig. S8**). In contrast, U5 is enriched near promoters in cluster P6, which are depleted of U6 elements (**Fig. S8**). These results further underscore the notion that these elements repress the genome in distinct ways.

While it is not surprising that U5 and U6 are enriched near genes with low expression since they are both enriched in repressive histone modifications, it is remarkable that these two chromatin signatures mark distinctly different populations of lowly expressed genes. One possibility is that U5 and U6 are present in different compartments of the nucleus. To test this, we examine the localization of these loci in lamina-associated domains (LADs), previously mapped in fibroblast cells and known to contain repressed genes and gene deserts. Indeed, more than 60% of U5 loci are in LADs (p_enrichment_<1E–300), compared to only 37.4% for U6 loci (p_depletion_ = 1.57E–10) (**Fig. 7B**). Taken together, these results suggest that U5 and U6 mark distinct domains of gene expression that may be explained by their enrichment in different nuclear compartments.

## Discussion

In this study, we survey the global landscape of commonly occurring chromatin signatures in the human genome. We recover known signatures at well-studied elements such as promoters and lesser-studied elements including enhancers. In addition, we find 7 distinct signatures spanning 47,874 genomic loci distal to known regulatory elements. We observe chromatin signatures marking exons and show at a higher resolution that the 5′ ends of exons are specifically modified by H3K36me3. Furthermore, we show that the enrichment level of this mark directly correlates with exonic expression, a result that had only been implied before. In addition, we recover two distinct chromatin modifications U1 and U2 marking exons in our genome-wide scan. While both are enriched in H3K36me3, U1 is uniquely enriched in H2BK5me1 and H4K20me1, which directly coincides with U1 marking early exons and U2 marking late exons.

A previous study by Kolasinska-Zwierz et al also observed that H3K36me3 marks exons in C. elegans and in mammals [8]. Here, we find that this histone modification is specifically enriched at the 5′ ends of exons and also weakly enriched eat 3′ ends of exons. Our results, together with findings by Kolasinska-Zwierz et al, implicate chromatin modifications in regulating splicing, a process until recently thought to be decoupled from transcription both physically and temporally. In yeast, H3K36me3 is deposited by the histone methyltransferase Set2, which is associated with the elongation form of RNA polymerase [40],[41]. The observation that H3K36me3 marks exons, a part of gene structure in the realm of splicing rather than transcription, implies that H3K36me3 may directly or indirectly regulate splicing.

A large body of work on splicing regulation has been focused on how sequence-specific proteins binding directly to pre-mRNAs affect splicing [42],[43]. But the static and highly degenerate natures of sequence elements associated with splicing leave unanswered the question of how cell-type specific splicing is achieved. However, recent discoveries physically linking RNA polymerase to the splicing machinery has shifted attention to the roles of the transcription machinery in regulating splicing [42],[44]. This has led to two models describing co-transcriptional splicing: a kinetic model and a recruitment model [42]. While both models emphasize spliceosome activity during transcription, neither fully explains how cell-type specific splicing is achieved. Our observations that distinct chromatin signatures are present at exons, and that different signatures are associated with either inclusion or exclusion from mature mRNAs, suggest a role of chromatin state in splicing regulation. One possibility is that the writing and reading of dynamic chromatin signatures may direct splicing events. While this model is attractive, further studies will be necessary to verify this hypothesis.

Identifying alternatively spliced exons *de novo* using chromatin signatures is an exciting possibility. A recent study has used the enrichment of H3K4me3 in conjunction with proximal enrichment of H3K36me3 to identify novel long non-coding RNAs [45], though H3K36me3 enrichment was used more as an indicator of elongation than of exon boundaries. But even if chromatin signatures can be used to detect alternative exons, because exons are transcribed it would be as cheaper, more efficient, and more reliable to employ techniques such as RNA-Seq to completely enumerate alternative exons *de novo* [46]. In the future as we approach completely mapping all histone modifications of the epigenome, one interesting possibility is that, like promoters and enhancers [3],[7], an exon chromatin signature marking poised but inactive exons may also exist. This could allow for identification of alternative exons needed for cellular response to stimuli.

We also recover several chromatin signatures enriched in repressive histone modifications marking distinct populations of repetitive elements. Surprisingly, these signatures are associated with different modes of gene repression. One possible explanation for this phenomenon is that U5 loci, which contain H3K9me2 and H3K9me3, are more highly enriched in nuclear lamina-associated domains than U6 loci. Thus the U5 chromatin signature may be specifically associated with LADs, while U6 is with other types of domains. It is possible that these two different types of chromatin domains correlate with distinct mechanisms of gene silencing, with H3K9-associated U5 domains being more permanently repressed than H3K9-free U6 domains.

These results show that studying the human genome on the basis of chromatin signatures is a useful method to cataloging regulatory elements in the genome in a global, unbiased, and systematic way. Future efforts to map chromatin modifications in the human genome may allow us to define more chromatin signatures marking novel regulatory elements or different functional specificities of known regulatory elements.

## Methods

**Data normalization.** Genome-wide distributions of histone modifications were obtained from Barski et al [12]. As in Hon et al [5], we filtered reads for uniqueness and redundancy, partitioned the genome into 100-bp bins, and counted reads in each bin. As the number of reads for each mark was highly variable, normalization was necessary to facilitate comparison. For each bin *i* and mark *h*, we normalized the number of reads in this bin *x_h,i_* as in [5]:
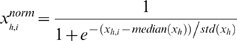



**Genome annotations.** Genome annotations were downloaded from the UCSC Genome Browser [37], human genome Build 36.1 (hg18 assembly). Gene definitions were given by the Refseq Genes [13] track. CpG island definitions were given by the “CpG Islands” track. Alternatively spliced exons were defined by entries in the “Alt Events” track labeled as “Cassette Exons”. The list of human loci conserved in a 28-way alignment with placental mammals was defined by the phastConsElements28wayPlacMammal table[24]. Repeat definitions were given by the RepeatMasker track [39], and lamina-associated domains mapped in Tig3 human lung fibroblasts [47] were defined by the “NKI LADs” track.

**Catalogs of regulatory elements.** Using previously published CTCF ChIP-Seq data [12], we obtained a list of 27,110 CTCF sites by running the Model-based Analysis of ChIP-Seq [27] software with default parameters and a p-value cutoff of 1E–5. We used normalized H3K4me1 and H3K4me3 profiles (as above) to predict enhancers as in Heintzman et al [6]. ROC analysis indicated that using a p-value cutoff of 0.1 gives optimal recovery (in terms of sensitivity and positive predictive value) of DNase I hypersensitive sites [23], corresponding to 32,237 predicted enhancers at least 2.5-kb from Refseq TSSs.

**Finding ChIP-enriched loci distal to known regulatory elements.** As in Hon et al [5], we identified regions of width 2-kb containing enrichment for histone modifications. We modeled the background distribution using 1% of the human genome as defined by the ENCODE regions and defined enriched regions as those significantly deviating (p = 0.0001) from the background. To remove redundancy, we removed any enriched locus closer than 2.5 kb to another enriched locus. We then removed loci within 2.5 kb to regulatory loci at promoters [13], gene 3′ ends [13], CTCF binding sites [12], DNase I hypersensitive sites [23], and sites having an enhancer chromatin signature [6].

**Finding chromatin signatures.** We searched for chromatin signatures of width 4-kb using ChromaSig [5] with a background prior *p_2A_* = 0.01 and a standard deviation factor *σ_another_* = 1.75. For loci with well-defined loci (gene 5′ ends, gene 3′ ends, CTCF binding sites, DNase I hypersensitive sites) we searched within a region ±500-bp around the sites, but for less-defined loci (predicted enhancers, ChIP-rich regions) we relaxed the search to a ±1-kb region. To focus only on the most frequently-occurring chromatin signatures, we analyzed only those clusters output having at least 500 loci and an average normalized enrichment greater than 0.25 for at least one modification.

**Chromatin signature significance.** For a given cluster of size N, we defined the motif *m_h,i_* to be the mean normalized enrichment of the aligned loci at a specified position *i* for modification *h*. Well-aligned motifs have higher values of enrichment. For each motif, we computed the score:
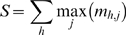



Higher values of S indicate more significant motifs. To assess significance of observing a motif spanning N loci with score S or greater, we randomly sampled 100 sets of clusters with random alignment offsets (within ±1 kb of the aligned sites) and orientations (positive or negative strand), computed S scores for each random set, and modeled the random distribution of S scores as a Guassian distribution to allow for calculation of significance. We performed this randomization either within loci in the same cluster as the original motif or over loci from all clusters.

**Heatmaps.** All heatmaps consist of normalized data over 100-bp bins (see above), and were visualized using Java TreeView [48].

**Expression data.** Transcript and exon expression data were measured in CD4+ T cells by Crawford et al [14] (GEO accession GSE4406) and Oberdoerffer et al [34] (GEO accession GSE11834), respectively. Both studies performed duplicate measurements on microarrays, and the expression data shown here is the average of the replicates.

**Randomization.** To determine enrichment for a given cluster, we compared to 100 random clusters. Each random cluster contains the same number of loci as the original cluster and follows the same chromosomal distribution. Random sampling is limited to bins containing ChIP-Seq reads.

**Statistical tests.** To assess significance of overlap with known genome annotations, we assume that the overlap statistics for 100 random clusters follows a Gaussian distribution. To assess significance of exon inclusion for marked versus unmarked exons, we use a two-sided Wilcoxon rank sum test to compare the median exon expression of the two sets. To assess that U5 and U6 are enriched near different classes of expressed genes, we use the paired two-sided Wilcoxon signed rank test to compare the enrichment profiles.

## Supporting Information

Figure S1 U1, U2, and U4 mark exon 5′ ends. An exon is unambiguously marked if it is the only exon within 1-kb of a genomic locus. We profiled chromatin enrichment relative to the 5′ ends of unambiguously marked exons for clusters (a) U1, (b) U2, (c), U3, and (d) U4. The top panels are heat maps representing the H3K36me3 enrichment in a 10-kb region surrounding the 5′ ends of unambiguously marked exons. The bottom panels represent the average profiles of the heat maps. U3 is the negative control.
(0.89 MB TIF)

Figure S2 U1 and U2 mark the 5′ ends of exons greater than 1-kb in length. An exon is unambiguously marked if it is the only exon within 1-kb of a genomic locus. We profiled chromatin enrichment relative to the 5′ ends of unambiguously marked exons of length >1-kb for clusters U1 and U2. The top panels are heat maps representing the H3K36me3 enrichment in a 10-kb region surrounding the 5′ ends of unambiguously marked exons. The bottom panels represent the average profiles of the heat maps. Only a small number of U3- and U4-marked unambiguous exons are larger than 1-kb, and so are not shown here.
(0.76 MB TIF)

Figure S3 Chromatin signatures associated with preferential inclusion and exclusion of exons into mature mRNAs. (a) Schematic of a gene containing an exon marked by a chromatin signature in pink and an unmarked alternatively spliced exon in green. After transcription and splicing, mature mRNAs either have one exon or the other. We compared exonic expression for marked exons in pink versus unmarked alternatively spliced exons in green for (b) U1, (c) U2, (d) U3, and (e) U4. The overlap is in brown. Wilcoxon rank sum p-values are indicated. Red p-values indicate enrichment of marked over unmarked exons, while green p-values indicate enrichment of unmarked over marked exons. U3 is the negative control.
(0.85 MB TIF)

Figure S4 Distinct chromatin signatures spanning predicted enhancers. On the basis of a previously published enhancer chromatin signature having strong H3K4me1 enrichment but weak H3K4me3 enrichment, we predicted 32,237 promoter-distal enhancers. Applying ChromaSig to these loci using the full panel of chromatin modifications mapped by Barski et al., we recovered 11 clusters. The heat map represents the enrichment of H2AZ, 20 histone modifications, CTCF, and RNA polymerase II in the 10-kb region surrounding each enhancer prediction. To organize these clusters visually, we performed hierarchical clustering on the average profiles using a Pearson correlation distance metric (left).
(3.73 MB TIF)

Figure S5 Distinct chromatin signatures spanning promoter-distal and enhancer-distal CTCF binding sites. We used MACS [10] to identify 27,110 CTCF binding sites from the Barski et al maps [5], 17,328 of which are distal to promoters and predicted enhancers. Applying ChromaSig to the chromatin modifications around these loci, we recovered 7 clusters. The heat map represents the enrichment of H2AZ, 20 histone modifications, CTCF, and RNA polymerase II in the 10-kb region surrounding each distal CTCF binding site. To organize these clusters visually, we performed hierarchical clustering on the average profiles using a Pearson correlation distance metric (left).
(1.75 MB TIF)

Figure S6 Distinct chromatin signatures spanning Refseq 3′ ends distal to Refseq promoters. Applying ChromaSig to the histone modifications near 16,703 Refseq gene 3′ ends that are distal to Refseq TSSs, we recover 12 clusters. The heat map represents the enrichment of H2AZ, 20 histone modifications, CTCF, and RNA polymerase II in the 10-kb region surrounding each Refseq gene 3′ end. To organize these clusters visually, we performed hierarchical clustering on the average profiles using a Pearson correlation distance metric (left).
(1.71 MB TIF)

Figure S7 Distinct chromatin signatures spanning DNase I hypersensitive sites. Previously, Boyle et al mapped 95,709 DNase I hypersensitive sites in CD4+ T cells, 31,824 of which are distal to Refseq TSSs, CTCF binding sites, and enhancer predictions. We applied ChromaSig to the chromatin modifications around these loci, recovering 13 clusters. The heat map represents the enrichment of H2AZ, 20 histone modifications, CTCF, and RNA polymerase II in the 10-kb region surrounding each distal DNase I hypersensitive site. To organize these clusters visually, we performed hierarchical clustering on the average profiles using a Pearson correlation distance metric (left).
(3.28 MB TIF)

Figure S8 Chromatin signatures of distal regulatory elements correlate with different classes of promoters. We partitioned the genome into CTCF-defined domains and counted the number of predicted enhancers and DNase I hypersensitive sites in each promoter-containing domain. To calculate enrichment, we compared to distributions of 100 sets of randomly placed loci (see Methods).
(0.72 MB TIF)

Figure S9 Distinct genomic distributions of chromatin signatures. The percentage each cluster within the 5′ and 3′ ends of genes (black), as compared to random sites (grey). The error bars indicate 1 standard deviation.
(0.19 MB TIF)

Figure S10 The distribution of H3K36me3 reads within exon and introns. The number of reads found within introns and exons, normalized by the total size of each.
(0.04 MB TIF)

Figure S11 The distribution of H3K36me3 reads at long exon 5′ and 3′ ends. The top panel shows the enrichment of H3K36me3 within 5-kb from (left) exon 5′ ends and (right) 3′ ends, for the longest 30,000 exons sorted by length (far right). The bottom panel is the average H3K36me3 enrichment profile of the shortest, middle, and longest third of exons from the top panel.
(1.41 MB TIF)

Table S1 Locations of clusters recovered when applying ChromaSig to Refseq promoters.
(0.31 MB TXT)

Table S2 Locations of clusters recovered when applying ChromaSig to predicted enhancers.
(0.51 MB TXT)

Table S3 Locations of clusters recovered when applying ChromaSig to CTCF binding sites.
(0.27 MB TXT)

Table S4 Locations of clusters recovered when applying ChromaSig to Refseq gene 3′ ends.
(0.25 MB TXT)

Table S5 Locations of clusters recovered when applying ChromaSig to DNase I hypersensitive sites.
(0.48 MB TXT)

Table S6 Locations of clusters recovered when applying ChromaSig to ChIP-enriched sites distal to Refseq promoters, Refseq gene 3′ ends, predicted enhancers, CTCF binding sites, and DNase I hypersensitive sites.
(0.80 MB TXT)

Table S7 Statistical significance of observed chromatin signatures. Significance for each cluster is calculated by comparing to random sets of clusters sampled from within the cluster or over all clusters.
(0.30 MB PDF)
