# Supplementary figures and images for: Discovery and Annotation of Functional Chromatin Signatures in the Human Genome
Source: PLoS Comput Biol. 2009 Nov 13;5(11):e1000566. doi: 10.1371/journal.pcbi.1000566 (PMC2775352; doi:10.1371/journal.pcbi.1000566)

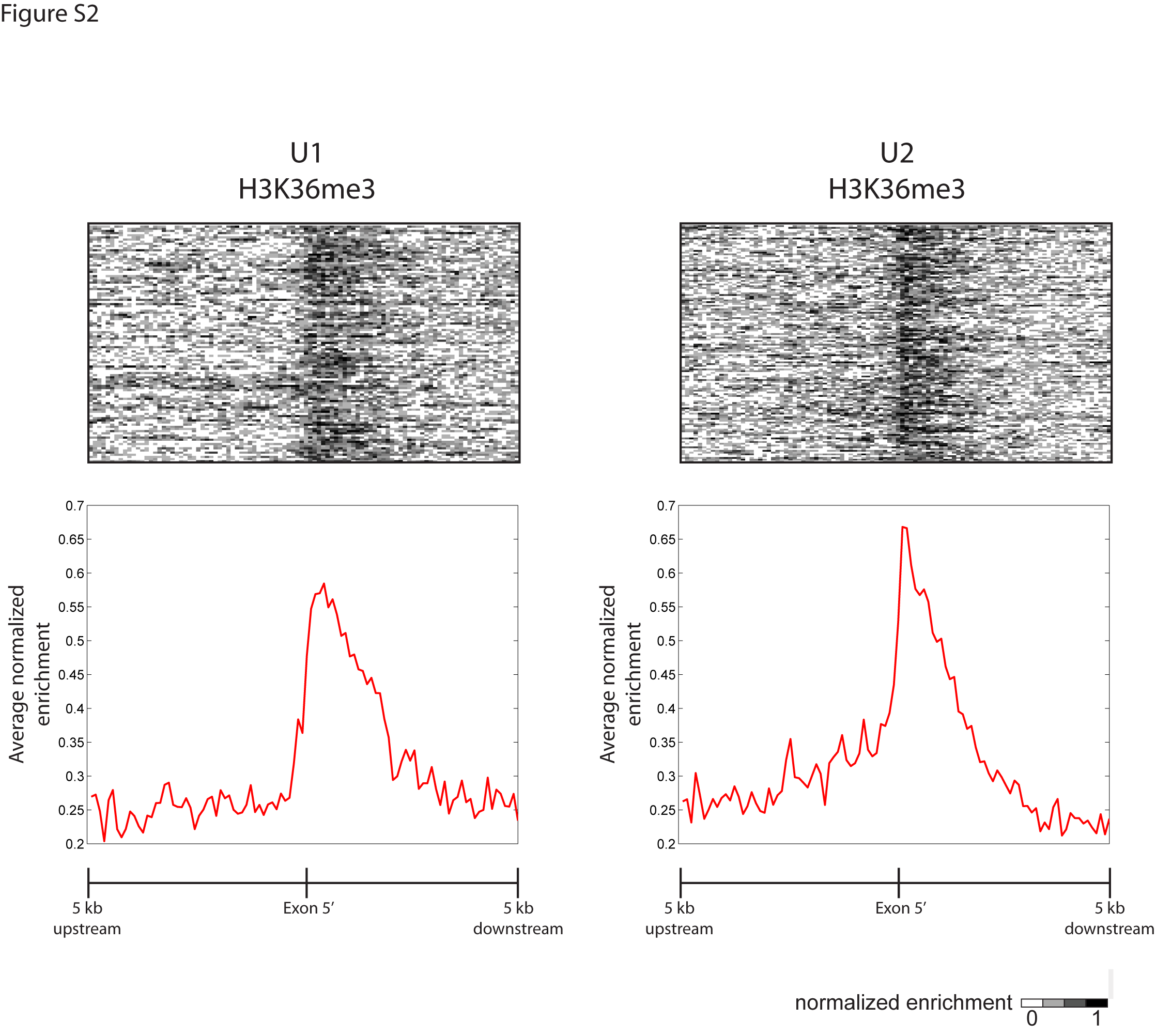

Supplement: Figure S2 — U1 and U2 mark the 5′ ends of exons greater than 1-kb in length. An exon is unambiguously marked if it is the only exon within 1-kb of a genomic locus. We profiled chromatin enrichment relative to the 5′ ends of unambiguously marked exons of length >1-kb for clusters U1 and U2. The top panels are heat maps representing the H3K36me3 enrichment in a 10-kb region surrounding the 5′ ends of unambiguously marked exons. The bottom panels represent the average profiles of the heat maps. Only a small number of U3- and U4-marked unambiguous exons are larger than 1-kb, and so are not shown here. (0.76 MB TIF) [file pcbi.1000566.s002.tif]

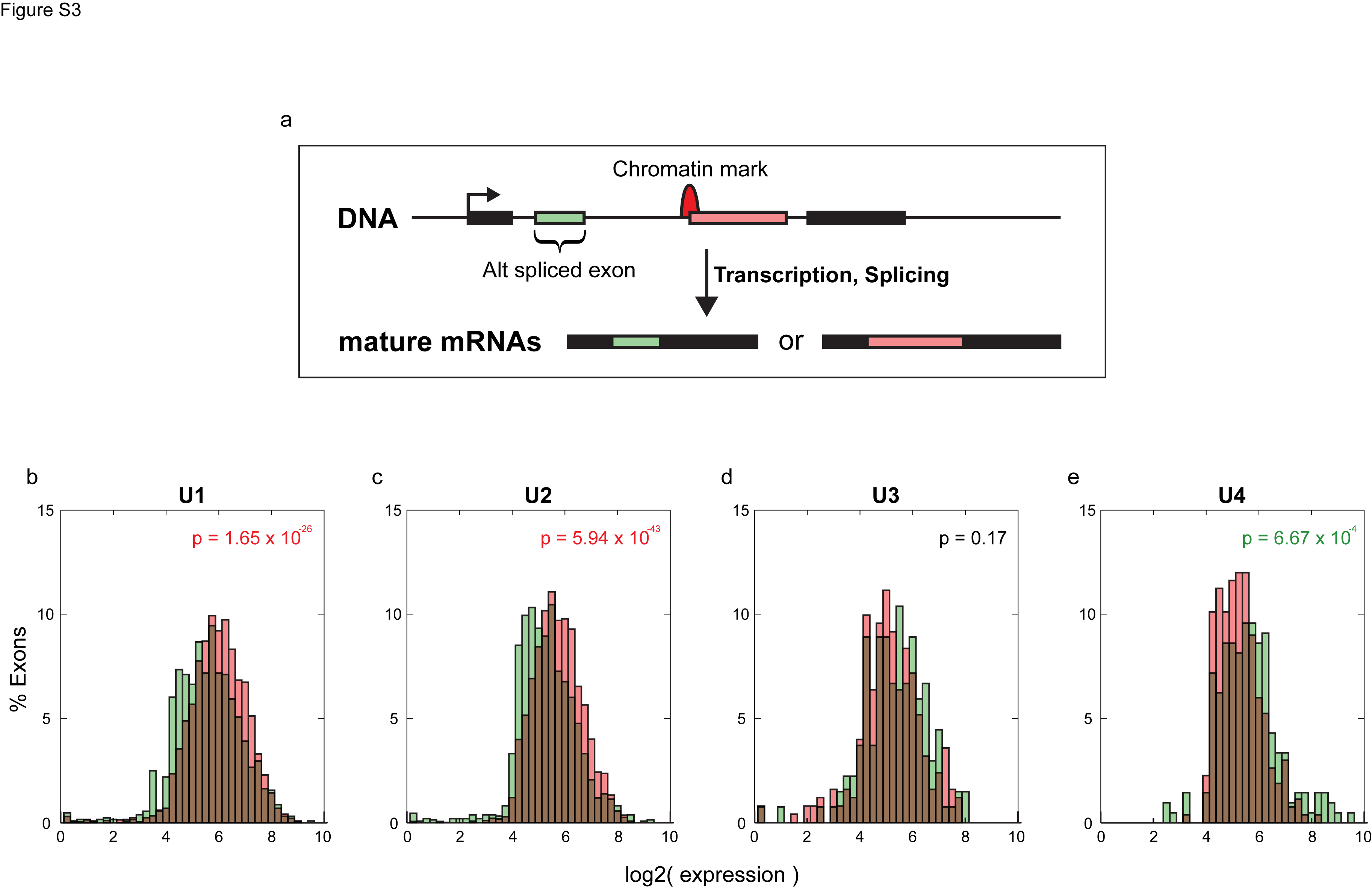

Supplement: Figure S3 — Chromatin signatures associated with preferential inclusion and exclusion of exons into mature mRNAs. (a) Schematic of a gene containing an exon marked by a chromatin signature in pink and an unmarked alternatively spliced exon in green. After transcription and splicing, mature mRNAs either have one exon or the other. We compared exonic expression for marked exons in pink versus unmarked alternatively spliced exons in green for (b) U1, (c) U2, (d) U3, and (e) U4. The overlap is in brown. Wilcoxon rank sum p-values are indicated. Red p-values indicate enrichment of marked over unmarked exons, while green p-values indicate enrichment of unmarked over marked exons. U3 is the negative control. (0.85 MB TIF) [file pcbi.1000566.s003.tif]

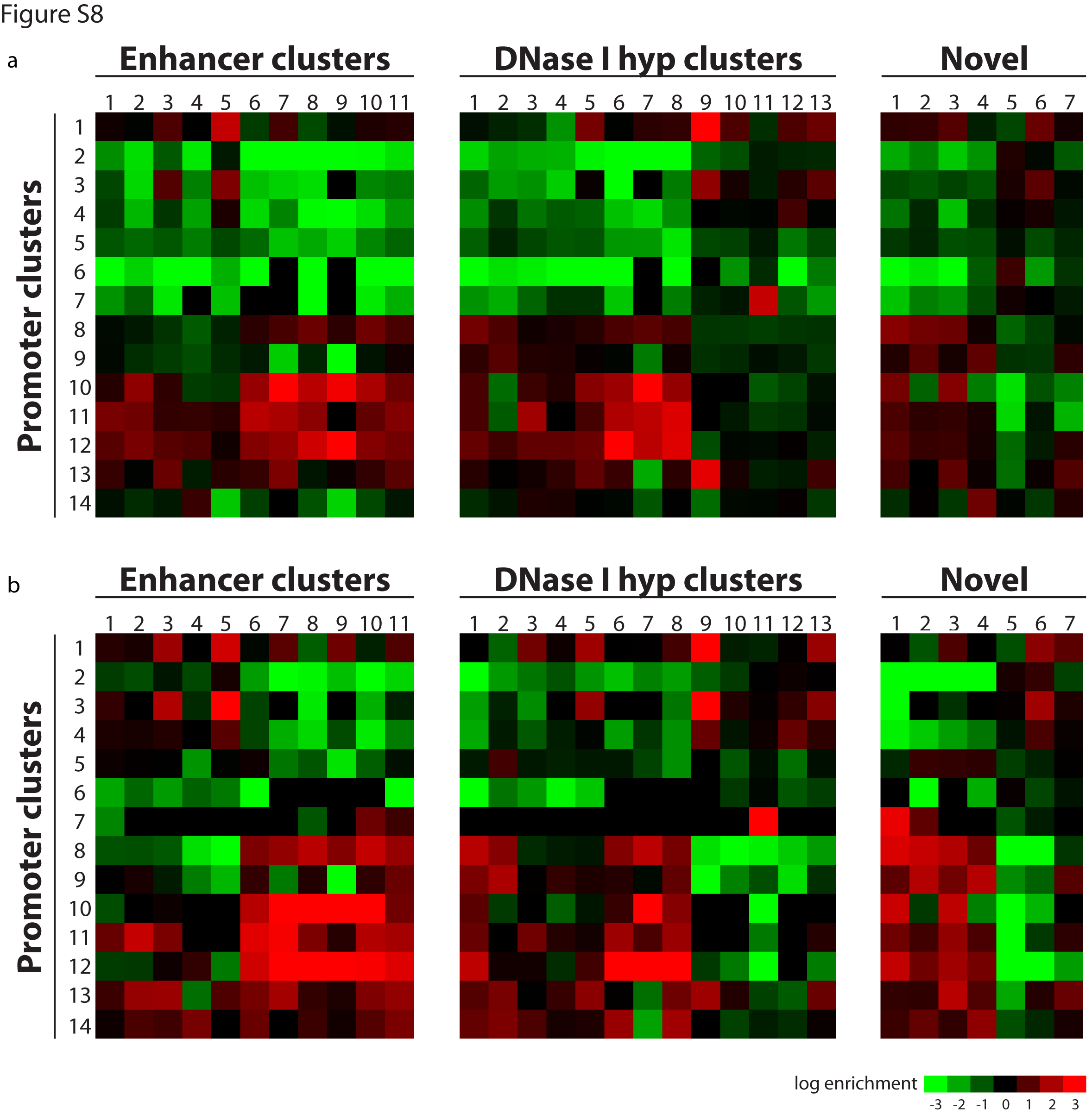

Supplement: Figure S8 — Chromatin signatures of distal regulatory elements correlate with different classes of promoters. We partitioned the genome into CTCF-defined domains and counted the number of predicted enhancers and DNase I hypersensitive sites in each promoter-containing domain. To calculate enrichment, we compared to distributions of 100 sets of randomly placed loci (see Methods). (0.72 MB TIF) [file pcbi.1000566.s008.tif]

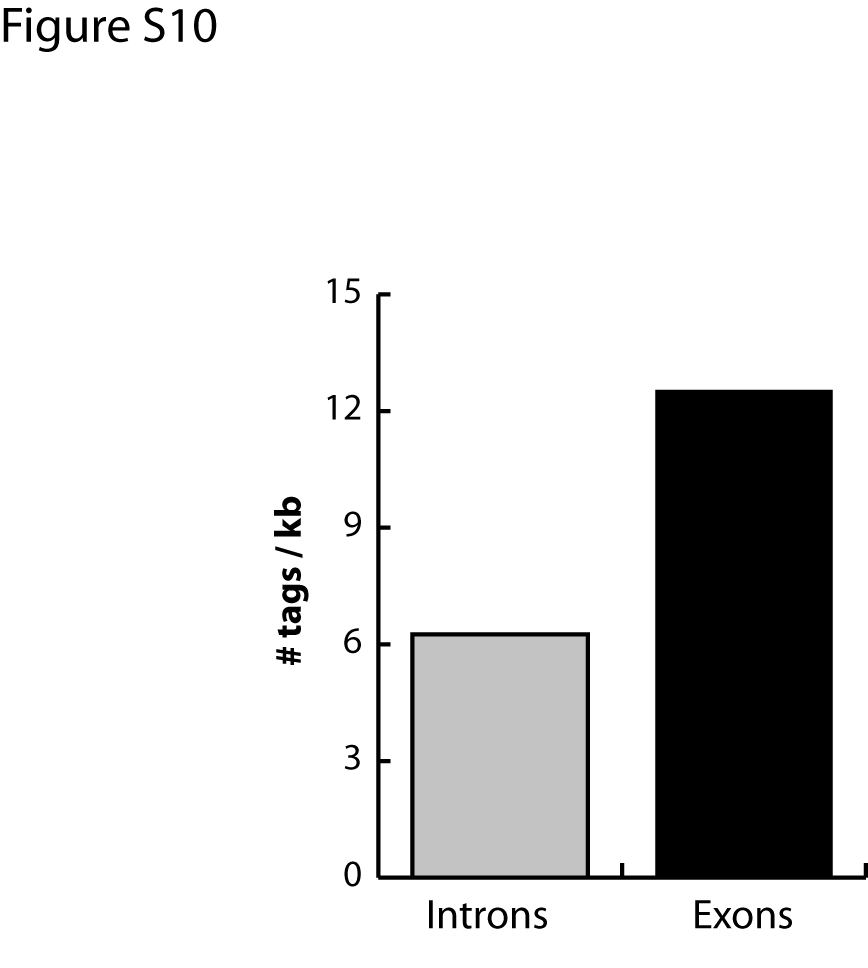

Supplement: Figure S10 — The distribution of H3K36me3 reads within exon and introns. The number of reads found within introns and exons, normalized by the total size of each. (0.04 MB TIF) [file pcbi.1000566.s010.tif]

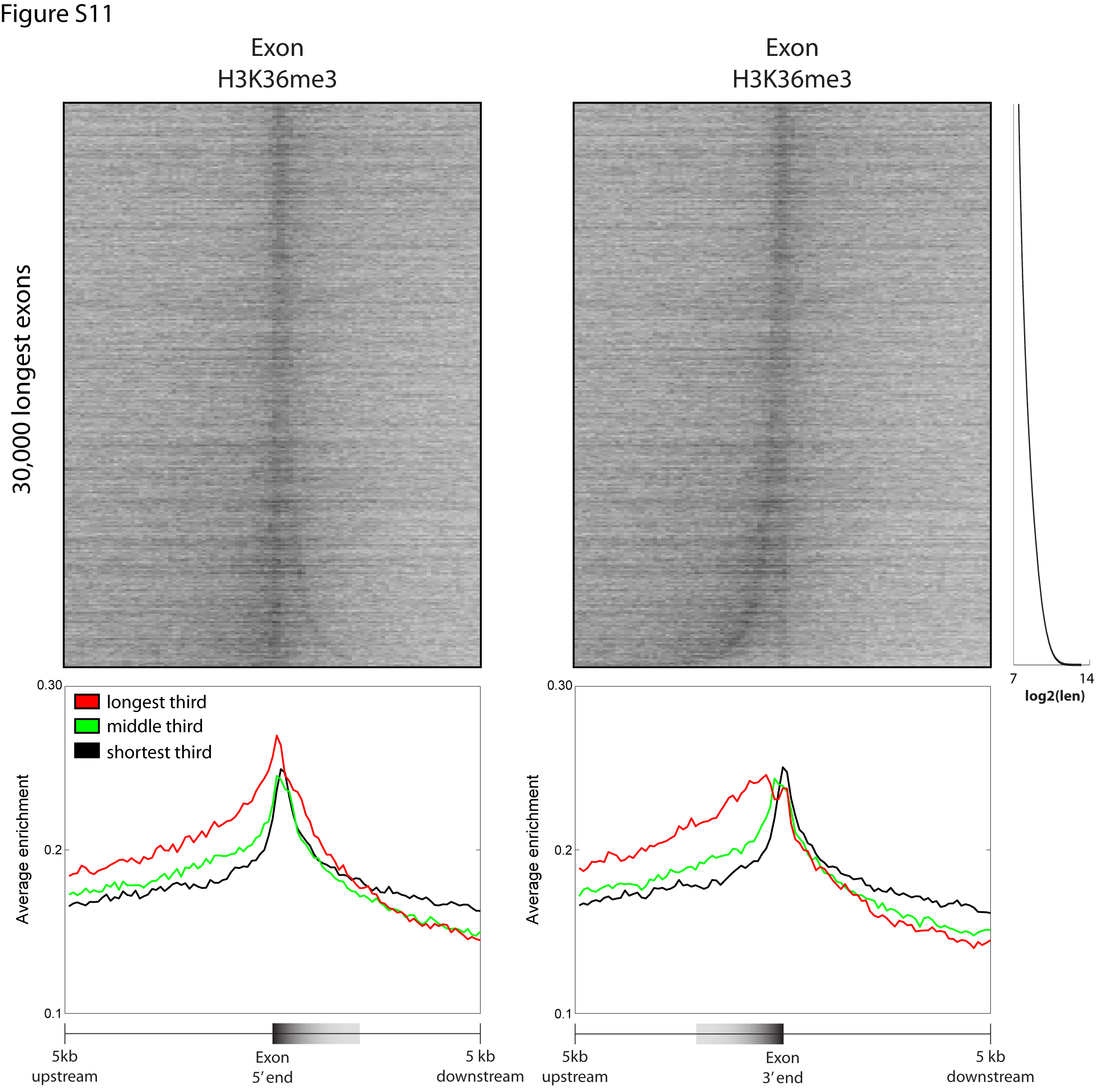

Supplement: Figure S11 — The distribution of H3K36me3 reads at long exon 5′ and 3′ ends. The top panel shows the enrichment of H3K36me3 within 5-kb from (left) exon 5′ ends and (right) 3′ ends, for the longest 30,000 exons sorted by length (far right). The bottom panel is the average H3K36me3 enrichment profile of the shortest, middle, and longest third of exons from the top panel. (1.41 MB TIF) [file pcbi.1000566.s011.tif]
