# Supplementary material for: Discovery and Annotation of Functional Chromatin Signatures in the Human Genome
Source: PLoS Comput Biol. 2009 Nov 13;5(11):e1000566. doi: 10.1371/journal.pcbi.1000566 (PMC2775352; doi:10.1371/journal.pcbi.1000566)
Supplement: Table S7 — Statistical significance of observed chromatin signatures. Significance for each cluster is calculated by comparing to random sets of clusters sampled from within the cluster or over all clusters. (0.30 MB PDF) [file pcbi.1000566.s018.pdf]

Table S7

		All clusters		Within cluster	
	**size**	**Z-score**	**p-val**	**Z-score**	**p-val**
**U1**	2845	57.46	< 1E-300	17.98	1.49E-72
**U2**	3742	34.61	9.53E-263	25.81	3.10E-147
**U3**	615	35.92	6.36E-283	14.06	3.32E-45
**U4**	961	36.60	1.52E-293	24.83	2.23E-136
**U5**	34368	17.99	1.20E-72	71.32	<1E-300
**U6**	4394	60.51	<1E-300	59.43	<1E-300
**U7**	949	8.72	1.38E-18	21.04	1.55E-98
